# The “Writing on the Wall Maneuver” Reveals the Dystonic Nature of Primary Writing Tremor

**DOI:** 10.1002/mdc3.14248

**Published:** 2024-11-09

**Authors:** Roberto Erro, Maria Chiara Malaguti, Alberto Morini, Christian Geroin, Michele Tinazzi

**Affiliations:** ^1^ Department of Medicine Surgery and Dentistry “Scuola Medica Salernitana”, Neuroscience section, University of Salerno Baronissi Italy; ^2^ Department of Neurology “Santa Chiara Hospital”, Azienda Provinciale per I Servizi Sanitari (APSS) Trento Italy; ^3^ Department of Surgery Dentistry, Paediatrics and Gynecology, University of Verona Verona Italy; ^4^ Department of Neurosciences Biomedicine and Movement Sciences, University of Verona Verona Italy

**Keywords:** task‐specificity, tremor, dystonia, writer's cramp

The concept of primary writing tremor (PWT) was first introduced by Rothwell et al in 1979 to describe a type of task‐specific tremor in which tremor predominantly occurs and interferes with handwriting.^1^ PWT is a rare form of tremor^2^ and in one of the largest series reported so far two types of PWT could be identified, type A or type B depending on whether tremor appeared during writing (type A: task‐induced tremor) or whilst writing and also on adopting the hand position normally used for writing (type B: positionally sensitive tremor).^1^ The nature of this entity has been disputed over the years with evidence supporting its relationship with either essential tremor (ET) or dystonic tremors (DT).^3^, ^4^, ^5^ The debate on what PWT represents has not been settled and, according to the new consensus on tremor classification, task‐specific tremors are classified separately from both ET and DT.

We have personally observed that in patients with tremulous task‐specific dystonia, writing on a wall ameliorates the dystonic posturing as well as their tremor (an exemplificative case with tremulous writer's cramp is shown in Video 1), a phenomenon that we did not encounter in patients with ET (Video 2). We, therefore, tested whether performing the same maneuver could ameliorate tremor in PWT and here report on two such patients in whom the “writing on the wall maneuver” led to a dramatic improvement of their tremor.

**Video 1:** A writer's cramp with clear index finger hyper‐extension and an additional tremor component is shown. The “writing on the wall maneuver” ameliorates both the dystonic movements and the tremor.

**Video 2:** In this essential tremor patient (who has both postural and kinetic tremor of the upper limbs) the “writing on the wall maneuver” fails to improve his tremor.

These two male patients aged 45 and 72 years, respectively, had a 1 to 4‐year duration of tremor that was only visible upon writing and not when performing any other activities. Their tremor did not appear simply when adopting the position used from writing, hence fulfilling the criteria for PWT type A. There were no signs of overt dystonia in the right arm or elsewhere, or any other neurological sign. In both cases, writing on a wall led to a dramatic improvement of their tremor (Video 3 and 4). We interpret the improvement as a result of the engagement of a different motor program used to accomplish the task, in keeping with a dystonic pathophysiological mechanism, although we cannot entirely exclude a contribution stemming from the change in the position of the arm. However, we do not believe the latter was the main factor explaining the improvement since our patients had a type A (task‐specific) rather than type B (positionally sensitive tremor) PWT.

**Video 3:** This patient has a tremor as soon as he initiates writing and not simply adopting the position used to write. There might be a hint of hyper‐extension of the first two fingers, but this was attributed to the tremor activity. No supporting features of dystonia such as over‐flow, mirroring or sensory trick were present. A marked reduction of the tremor is observed during the “writing on the wall maneuver” as also shown by the comparison between the writing outputs.

**Video 4:** This patient also has a tremor that appears only during writing and not during other tasks. A marked reduction of the tremor is observed during the “writing on the wall maneuver”.

“The writing on the wall” is an idiom that suggests a portent of doom, based on the story of Belshazzar's feast in the Book of Daniel (Fig. 1). We suggest that the “writing on the wall maneuver” could disclose the dystonic nature of PWT. Larger and longitudinal observations on PWT patients are required to assess: (1) whether the “writing on the wall maneuver” could predict the development of overt dystonia; (2) if it improves tremor also in non task‐specific dystonia of the upper limb; and (3) its specificity against ET and ET‐plus, especially in the presence of soft dystonic signs.

**Figure 1:**
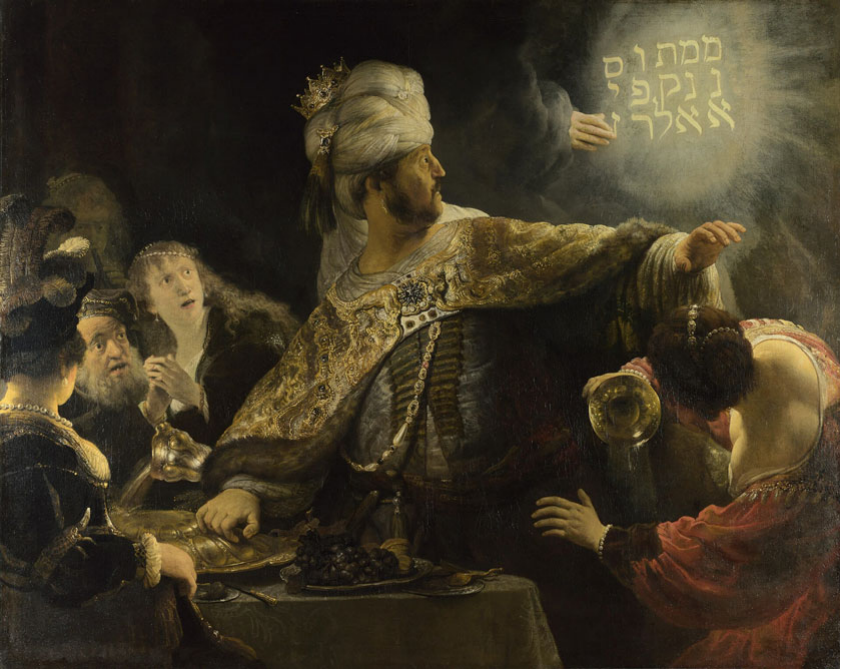
Rembrandt's depiction of the biblical account of Belshazzar seeing “the writing on the wall.”

## Author Roles

(1) Conception and design of the study, or acquisition of data, or analysis and interpretation of data; (2) Drafting the article or revising it critically for important intellectual content; (3) Final approval of the version to be submitted.

R.E.: 1, 2, 3.

M.C.M.: 2, 3.

A.M.: 2, 3.

C.G.: 2, 3.

M.T.: 2, 3.

## Disclosures

**Ethical Compliance Statements:** This was work has been approved by the ethic committee of the coordinator center and written informed consent was obtained from the patients prior to their enrolment. We confirm that we have read the Journal's position on issues involved in ethical publication and affirm that this work is consistent with those guidelines.

**Funding Sources and Conflict of Interest:** This study did not receive any funding nor was performed as part of the employment of the authors. The authors state explicitly that there are no conflicts of interest in connection with this article.

**Financial disclosures for the previous 12 months:** R.E. receives royalties from publication of *Case Studies in Movement Disorders—Common and Uncommon Presentations* (Cambridge University Press, 2017) and of *Paroxysmal Movement Disorders* (Springer, 2020). He has received consultancies from Ipsen and Jazz Pharma and honoraria for speaking from the International Parkinson's Disease and Movement Disorders Society. All other authors have nothing to disclose.

## Data Availability

The data that support the findings of this study are available from the corresponding author upon reasonable request.

## References

[1] Bain PG , Findley LJ , Britton TC , Rothwell JC , Gresty MA , Thompson PD , Marsden CD . Primary writing tremor. Brain 1995;118(Pt 6):1461–1472. 10.1093/brain/118.6.1461. 8595477

[2] Erro R , Pilotto A , Esposito M , et al. The Italian tremor network (TITAN): rationale, design and preliminary findings. Neurol Sci 2022;43(9):5369–5376. 35608737 10.1007/s10072-022-06104-w PMC9385818

[3] Erro R , Reich SG . Rare tremors and tremors occurring in other neurological disorders. J Neurol Sci 2022;15(435):120200. 10.1016/j.jns.2022.120200. 35220114

[4] Latorre A , Rocchi L , Batla A , Berardelli A , Rothwell JC , Bhatia KP . The signature of primary writing tremor is dystonic. Mov Disord 2021;36(7):1715–1720. 10.1002/mds.28579. 33786886

[5] Pandey S , Datta A . A primary writing tremor is a form of dystonic tremor: is the debate settled? Mov Disord 2021;36(8):1995–1996. 34409691 10.1002/mds.28696

